# The association between cognitive impairment, gait speed, and Walk ratio

**DOI:** 10.3389/fnagi.2023.1092990

**Published:** 2023-05-18

**Authors:** Mari Kalland Knapstad, Ingvill Naterstad, Bård Bogen

**Affiliations:** ^1^Department of Health and Function, Western Norway University of Applied Sciences, Bergen, Hordaland, Norway; ^2^Department of Otorhinolaryngology & Head and Neck Surgery, Haukeland University Hospital, Bergen, Hordaland, Norway; ^3^Department of Global Public Health and Primary Care, University of Bergen, Bergen, Hordaland, Norway; ^4^Department of rehabilitation services, Haraldsplass Deaconal Hospital (HDS), Bergen, Hordaland, Norway

**Keywords:** Walk ratio, gait speed, cognitive impairment, older adults, motor control

## Abstract

**Background:**

Gait speed has been found to be associated with cognitive function. However, gait speed is an unspecific measure that may not be informative about gait patterns. The Walk ratio (step length divided by step frequency) can be measured without specialized equipment, and has been suggested as an indicator of central gait control. However, the association with cognitive function is unknown.

**Research question:**

Is there a relationship between Walk ratio and cognitive function, and gait speed and cognitive function?

**Methods:**

This was a systematic literature review of studies where spatiotemporal gait parameters was reported in populations with cognitive impairment. The search was performed through PubMed, PEDro, AMED, Cochrane, Embase, MEDLINE, and PsycINFO. The studies had to contain either the Walk ratio, or report average step length and average step frequency. In the latter case, the average step length was divided by the average step frequency. The studies also had to report gait speed and the minimal mental state examination (MMSE). Studies testing patients on treadmills or that did not state the exclusion of patients with neurologic or orthopedic diseases, possible affecting gait ability, were excluded.

**Results:**

A total of 24 studies were included, consisting of 909 patients with cognitive impairment and 4,108 healthy controls. The patient group had a lower Walk ratio (mean difference 0.07, *p* ≤ 0.001) and gait speed (mean difference 0.26, *p* ≤ 0.001) than the healthy controls. Using linear regression models, we found an association between the MMSE and the Walk ratio (*R*^2^ = 0.29, *p* < 0.001) and gait speed (*R*^2^ = 0.41, *p* < 0.001) in separate, unadjusted models. In a final model with Walk ratio, gait speed and age, Walk ratio was not significantly associated with MMSE, while gait speed was.

**Significance:**

Our results suggest that preferred gait speed may be preferable to the Walk ratio when assessing older adults with cognitive impairment.

## Background

The step length divided by the step frequency is often referred to as the Walk ratio, and this measure has seen increasing interest in clinical and research communities. The Walk ratio is assumed to be an invariant feature of walking, meaning that the relationship between how long and how many steps we take is constant at different gait speeds. This ratio tends to be around 0.6 cm/step/min in healthy people (Sekiya et al., 1996; Zijlstra et al., 2008; Bogen et al., 2018). Deviations from a value of around 0.6 cm/step/min can therefore potentially be interpreted as indicative of pathology. The Walk ratio has been called an indicator of automaticity of gait (Bogen et al., 2018) and of central gait coordination (Egerton et al., 2011), suggesting that for example degenerative brain disease is expressed through a change in the gait rhythm. Rota and co-authors have used the Walk ratio in people with multiple sclerosis, and found that it was lower in patients than controls, and suggest that it may be a summary measure of neuromotor control of gait (Rota et al., 2011). Gait speed has already been established as a very powerful indicator of different pathologies (Abellan van Kan et al., 2009; Studenski et al., 2011), including dementia, and subjective and mild cognitive impairment (Buracchio et al., 2010; Verghese et al., 2014; Hackett et al., 2018; Knapstad et al., 2019). Gait speed is well established in research as well as in many clinical centers and is arguably a little easier to measure than the Walk ratio. However, gait speed may not be informative about *how* the person walks. It is conceivable that a person can walk slowly with a steady and safe gait, and quickly with unstable and variable gait. Slow gait speed is associated with for example both high and low gait variability (between-stride fluctuations) (Moe-Nilssen and Helbostad, 2005; van Swearingen et al., 2017; Bogen et al., 2019). However, to measure gait variability, specialized equipment is required, and measurements of gait quality that can be done without specialized equipment could be useful in clinical practice.

Given the accumulating evidence that there is an association between gait and cognition (Buckley et al., 2019), it is conceivable that the Walk ratio may be an easily administered gait quality measure, that may be even more closely associated with cognitive impairment than gait speed. However, we have found no studies that investigate this.

The aim of this study is therefore to investigate how gait speed and the Walk ratio are associated with cognitive function, as measured with the Mini Mental State Examination (MMSE), in previously published research. We hypothesize that the Walk ratio has a closer association with MMSE than gait speed, as it is informative about gait quality, and as it has been suggested as informative about central gait control.

## Methods

### Research design

Because no studies have explored Walk ratio in a population with cognitive impairment, an exploratory literature search for studies examining gait function in this population was conducted. The results were narrowed down to studies containing the variables needed to calculate Walk ratio of the participants in the studies.

### Search strategy

**Figure 1:**
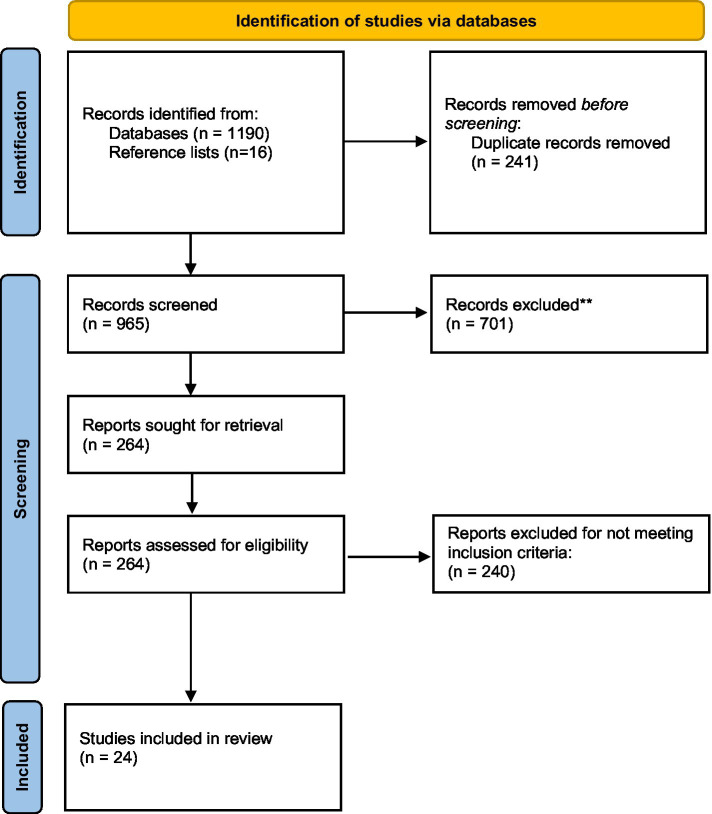
Flow chart of the included studies.

The literature search for this article was performed using PubMed, PEDro, AMED, Cochrane, Embase, MEDLINE and PsycINFO with assistance from an experienced librarian (last search date: 2nd of February 2021). The search terms were as follows: (step length OR stride length) AND (cadence OR step frequency OR gait OR Walk speed OR velocity OR Walk ratio) AND (dement* OR Alzheimer* OR mild cognitive impairment). The search terms were used as mesh terms or text words and were adjusted for the different databases. The full search strategy is available in the Supplementary Datasheet. We had no restrictions regarding research design, however; unpublished studies and abstracts were not included. Also, systematic reviews were not included. Languages were restricted to English. Article references were screened for potentially relevant studies, resulting in 16 additional articles. The PRISMA 2020 Flow Diagram (Page et al., 2021) was used to illustrate the selection process of the studies (Figure 1).

### Inclusion and exclusion criteria

To be included, participants had to be transparently classified as having cognitive impairment. For comparison across studies, they also had to be scored using the Mini Mental State Examination (MMSE) (Folstein et al., 1975). No age limit was used for inclusion or exclusion, and both men and women were included. Studies of both cross-sectional, longitudinal and experimental designs were included. Further, only studies reporting the Walk ratio, or step length and step frequency, were included. To make the gait assessment comparable, only studies using steady-state walking were included, meaning that acceleration and deceleration phases were not included as part of the walk. Further, only studies using overground walking were included, excluding studies using treadmill walking. Studies that included patients with neurological or orthopedic conditions that would affect their gait were not included. The included studies were not assessed for methodological quality.

### Variables and data collection

Some of the studies had several groups like control groups and interventions groups, resulting in a higher number of groups than the number of included studies. Variables extracted from the studies were baseline MMSE-score, gait speed (meters per second), age and Walk ratio from each group, thus the mean of each group was collected. In studies reporting step length and step frequency but not the Walk ratio, the Walk ratio was calculated by dividing the mean step length of the sample by the mean step frequency. Healthy control groups were included as long as they met the criteria for inclusion.

### Statistical analysis

The following variables were computed: MMSE-scores, gait speed and Walk ratio scores and age. We retrieved and calculated Walk ratio from the average step frequency and step length. The relationship between MMSE, gait speed, Walk ratio and age were examined with simple and multiple linear regressions. The following regression analyses were made: (i) simple linear regression with MMSE as dependent variable, and age, gait speed and Walk ratio as independent variables respectively, (ii) adjusted analyses with age as an independent variable together with gait speed and Walk ratio, and (iii) fully adjusted analysis with age, gait speed and Walk ratio in the same model. Multicollinearity was assessed by inspecting the Variance Inflation Factor (VIF) and tolerance. VIF over 10 and tolerance below 0.1 were considered signs of multicollinearity (Pallant, 2020). Independent t-tests were used to compare means between controls and people with cognitive impairment, please not that these means represent the means of the baseline values of the included articles, and are therefore means of means. The alpha level was set to 0.05. Statistics were performed in Statistics Package for Social Science (SPSS) 25.0 for Windows.

## Results

The initial database search yielded 1,206 studies. After removing of duplicates (*n* = 241) and screening titles (*n* = 965), 264 full text articles were assessed for eligibility. Two hundred and forty of these were excluded, and 24 articles were included for analysis (Table 1).

**Table 1:** List of all included studies (*n* = 24).

	Year	Journal	Design	*N*	Population	MMSE*	Age	Gait speed (m/s)	Cadence (steps/min)	Step length (cm)	Walk ratio (cm/step/min)	Country	% Female
Healthy controls													
Cosentino et al.	2020	Alzheimer Dis Assoc Disord 2020; 34	Cross sectional	43	Normal controls	29	72	0.73	86.00	39.00	0.45	IT	58
De Cock et al.	2017	PLoS ONE 2017; 12(6)	Cross sectional	78	Normal controls	29	75	1.05	108.60	58.00	0.53	BEL	54
Doi et al.	2019	JAMDA 20 (2019)	Prospective study	3.766	No dementia	26	72	1.26	122.00	62.00	0.51	JPN	53
Goyal N et al.	2019	Int J Rehab Res 2019; 42	Cross sectional	8	Normal controls	29	73	1.10	115.10	56.60	0.49	US	63
Gras LZ et al.	2015	J Ger Phys Ter 2015; 38(1)		13	Normal controls	29	73	1.49	116.10	77.00	0.66	US	23
Ijmker T & Lamoth C J	2012	Gait & Posture 35 (2012)	Cross sectional	14	Older controls	29	80	1.40	112.14	61.00	0.54	NL	14
				12	Younger controls	29	64	1.19	112.40	64.00	0.57		25
McGough EL et al.	2018	Am J Phys Med Rehabil 2018;97(4)	Cross sectional	23	Normal controls	29	83	1.09	112.80	58.50	0.52	US	78
Montero-Odasso M et al.	2012	Arch Phys Med Rehabil 2012;93	Cross sectional	26	Normal controls	30	72	1.34	115.60	70.00	0.61	CA	88
Muir SW et al.	2012	Gait & Posture 35 (2012)	Cross sectional	22	Normal controls	30	71	1.36	114.58	71.00	0.62	CA	88
Nadkarni N et al.	2009	Gait & Posture 30 (2009)	Cross sectional	34	Normal controls	29	74	1.19	109.00	65.00	0.60	CA	45
Nadkarni N et al.	2009	Dement Geriatr Cogn Disord 2009;28	Correlation study	15	Normal Controls	28	76	1.11	106.00	62.00	0.58	CA	
				18	Normal controls	29	69	1.27	112.00	69.00	0.62		
Silva FO et al.	2020	Exp Gerontol 131 (2020)	Cross sectional	17	Normal controls	29	71	1.26	113.00	58.00	0.51	BR	100
Wittwer JE et al.	2010	Gait & Posture 32 (2010)	Longitudinal study	19	Normal controls	29	80	1.18	111.00	64.00	0.58	AUS	47
Cognitive impairment													
Cedervall Y et al.	2014	Gait & Posture 39 (2014)	Longitudinal study	21	Mild Alzheimer’s disease	25	72	1.14	110.32	62.00	0.56	SWE	52
				21	Mild Alzheimer’s disease *	22	73	1.10	110.00	60.00	0.55		52
				21	Mild Alzheimer’s disease*	21	74	1.01	106.32	57.00	0.54		52
Cosentino E et al.	2020	Alzheimer Dis Assoc Disord 2020;34	Cross sectional	43	Mild cognitive impairment	25	72	0.57	73.40	33.00	0.45	IT	61
De Cock AM et al.	2017	PLoS ONE 2017;12(6)	Cross sectional	96	Moderate dementia	15	83	0.78	100.00	45.00	0.45	BEL	74
de Melo Coelho FG et al.	2013	Gait & Posture. 39(4)	Intervention study	14	Mild and Moderate Alzheimer’s disease	20	78	0.79	103.04	46.00	0.45	BR	N.R.*
de Melo Coelho FG et al.	2012	Aging Neuropsych Cog. 19(5)	Cross sectional	12	Mild Alzheimer’s disease	22	76	0.78	99.60	47.00	0.47	BR	N.R.*
Doi T et al.	2019	JAMDA 20 (2019)	Prospective study.	245	Incident dementia	24	77	1.10	120.00	55.00	0.46	JPN	54
Doi T et al.	2012	Arch Gerontol Ger 56 (2013)	RCT	25	Amnestic mild cognitive impairment	27	75	1.10	115.79	57.00	0.49	JPN	46
Goyal N et al.	2019	Int J Rehab Res 2019;42	Cross sectional	8	Mild cognitive impairment	19	78	0.80	97.70	48.80	0.50	US	75
Gras LZ et al.	2015	J Ger Phys Ter 2015;38(1)	Cross sectional	13	Very mild Alzheimer’s disease	25	73	1.07	103.55	62.00	0.60	US	23
Ijmker T & Lamoth CJ	2012	Gait & Posture 35 (2012)	Cross sectional	15	Alzheimer’s disease and fronto temporal dementia	20	82	0.67	100.84	40.00	0.40	NL	12
Kim JS et al.	2017	EGEM 8 (2017)	Intervention study	15	Mild to moderate Alzheimer’s disease	14	80	0.53	95.21	33.15	0.35	KR	N.R.*
McGoughEL et al.	2018	Am J Phys Med Rehabil 2018;97(4)	Cross sectional	23	Amnestic mild cognitive impairment	27	85	0.99	110.00	54.00	0.49	US	73
Montero-Odasso M et al.	2009	J NeuroEngineer Rehab 2009. 6:35	Reliability study	13	Mild cognitive impairment	28	77	1.19	108.38	65.88	0.61	CA	54
Montero-OdassoM et al.	2012	Arch Phys Med Rehabil 2012;93	Cross sectional	43	Mild cognitive impairment	28	75	1.11	107.60	62.00	0.58	CA	54
Muir SW et al.	2012	Gait & Posture 35 (2012)	Cross sectional	29	Mild cognitive impairment	28	74	1.16	111.42	62.00	0.56	CA	59
				23	Alzheimer’s disease	24	78	1.11	108.72	61.00	0.56		61
Nadkarni NK et al.	2009	Gait & Posture 30 (2009)	Cross sectional	40	Alzheimer’s disease	25	74	0.99	101.00	59.00	0.58	CA	55
Nadkarni NK et al.	2009	Dement Geriatr Cogn Disord 2009;28	Corrolation study	21	Alzheimer’s disease	25	77	0.96	102.00	56.00	0.55	CA	
Schwenk M et al.	2014	BMC Geriatrics 2014 14:73	RCT	20	Milde to moderate dementia	21	80	1.33	137.14	58.29	0.43	GER	65
Silva FO et al.	2019	Exp Gerontol 131 (2020)	Cross sectional	23	Alzheimers’s disease	21	78	0.96	95.60	52.00	0.54	BR	52
				23	Mild cognitive impairment	29	77	1.21	108.50	58.00	0.53		61
van den Elsen GAH et al.	2016	J Psychopharmacology 2017 31(2)	RCT	17	Alzheimer’s disease	19	77	0.92	99.70	55.37	0.56	NL	17
Wittwer J et al.	2008	Gait & Posture 28 (2008)	Reliability study	20	Alzheimer’s disease	22	81	1.06	106.47	59.70	0.56	AUS	50
Wittwer J et al.	2013	Gait & Posture 38 (2013)	Reproducibility study	16	Alzheimer’s disease	21	81	1.00	104.70	57.35	0.55	AUS	63
Wittwer J et al	2014	Gait & Posture 39 (2014)	Cross sectional	30	Alzheimer’s disease	21	80	1.12	109.60	60.70	0.55	AUS	50
Wittwer J et al	2010	Gait & Posture 32 (2010)	Longitudinal study	11	Mild Alzheimer’s disease	25	80	1.12	105.90	64.00	0.60	AUS	50

Twenty four studies were included in the review (Wittwer et al., 2008, 2010, 2013, 2014; Montero-Odasso et al., 2009, 2012; Nadkarni et al., 2009; Coelho et al., 2012, 2013; Ijmker and Lamoth, 2012; Muir et al., 2012; Doi et al., 2013, 2019; Cedervall et al., 2014; Schwenk et al., 2014; Gras et al., 2015; De Cock et al., 2017; Kim et al., 2017; van den Elsen et al., 2017; McGough et al., 2018; Goyal et al., 2019; Cosentino et al., 2020; de Oliveira et al., 2020), of these 13 studies included control groups with participants without cognitive impairment (Nadkarni et al., 2009; Wittwer et al., 2010; Ijmker and Lamoth, 2012; Montero-Odasso et al., 2012; Muir et al., 2012; Gras et al., 2015; De Cock et al., 2017; McGough et al., 2018; Doi et al., 2019; Goyal et al., 2019; Cosentino et al., 2020; de Oliveira et al., 2020). In total, 909 patients with cognitive impairment were included, and 4,108 healthy controls without cognitive impairment. The average MMSE for the cognitively impaired was 22.64 (SD 3.92), and 28.76 (SD 0.75) for the healthy controls.

Three of the included studies had longitudinal design (Wittwer et al., 2010; Cedervall et al., 2014; Doi et al., 2019), 13 were cross-sectional studies (Nadkarni et al., 2009; Coelho et al., 2012; Ijmker and Lamoth, 2012; Montero-Odasso et al., 2012; Muir et al., 2012; Wittwer et al., 2014; Gras et al., 2015; De Cock et al., 2017; McGough et al., 2018; Goyal et al., 2019; Cosentino et al., 2020; de Oliveira et al., 2020), five were intervention studies (Coelho et al., 2013; Doi et al., 2013; Kim et al., 2017; van den Elsen et al., 2017) and three were reliability studies (Wittwer et al., 2008; Montero-Odasso et al., 2009; Wittwer et al., 2013). Due to the differing designs, no quality assessment of the studies was performed. Two studies (McGough et al., 2018; de Oliveira et al., 2020) explicitly stated that they followed the STROBE-checklist (von Elm et al., 2007). Six of the studies were done in Europe (Ijmker and Lamoth, 2012; Cedervall et al., 2014; Schwenk et al., 2014; De Cock et al., 2017; van den Elsen et al., 2017; Cosentino et al., 2020), eight in United States and Canada (Montero-Odasso et al., 2009, 2012; Nadkarni et al., 2009; Muir et al., 2012; Gras et al., 2015; McGough et al., 2018; Goyal et al., 2019), four in Australia (Wittwer et al., 2008, 2010, 2013, 2014), three in Latin America (Coelho et al., 2012, 2013; de Oliveira et al., 2020) and three in Asia (Doi et al., 2013, 2019; Kim et al., 2017). Some of the included studies had more than one group of cognitive impairment. The studies included nine groups with Mild Cognitive Impairment (*n* = 207, average MMSE 26.23), one group with very mild Alzheimer’s Disease (*n* = 13, average MMSE 24.8), four groups with mild Alzheimer’s Disease (*n* = 86, average MMSE 22.9), three groups with mild to moderate Alzheimer’s Disease (*n* = 49, average MMSE 18.03), two groups with moderate dementia (*n* = 104, average MMSE 15.8), eight groups with Alzheimer’s Disease without specified severity (*n* = 190, average MMSE 22.24), one group with Alzheimer’s Disease and Frontotemporal Dementia (*n* = 15, average MMSE 19.6) and one study with incident dementia (*n* = 245, average MMSE 24.3). All studies specified which criteria they used for establishing cognitive impairment: For MCI, objective and subjective memory impairment, with largely preserved function and intellectual ability was used (Petersen and Negash, 2008). Alzheimer’s Disease was established using DSM-IV-TR, NINCDS-ARDRA, or ICD-10 and DSM-5.

The number of participants with cognitive impairment in the studies varied between 13 and 245. The average age was approximately 77 years, and none of the included studies had an average age of participants that was lower than 72 years. None of the included studies reported the Walk ratio directly, and we divided the average step lengths by the average step frequencies to calculate the Walk ratio. The average gait speed and Walk ratio for the participants with cognitive impairment was 0.98 m/s and 0.52 cm/step/min, respectively. The individuals in the control group had an average gait speed of 1.20 m/s and an average Walk ratio of 0.56 cm/step/min (Table 2). This difference was statistically significant, and suggest that the persons with cognitive impairment walked slower and with a more cautious gait pattern. Also, the cognitively impaired participants were significantly older than the healthy controls.

**Table 2:** Means of mean values of the included studies on healthy controls and on cognitive impairment.

	Healthy controls (13 studies, *n* = 4,108)		Cognitive impairment cognitive impairment (24 studies, *n* = 909)		
	Mean	SD	Mean	SD	Value of p**
Walk ratio (cm/step/min)	0.56	0.06	0.52	0.06	0.022
Gait speed (m/s)	1.20	0.18	0.99	0.19	≤0.001
Age	73.56	4.61	77.38	3.34	0.003
MMSE* (0–30)	28.76	0.73	22.64	3.92	≤0.001

*MMSE, minimal mental state examination; **differences analyzed using independent sample *t*-tests.

**Figure 2:**
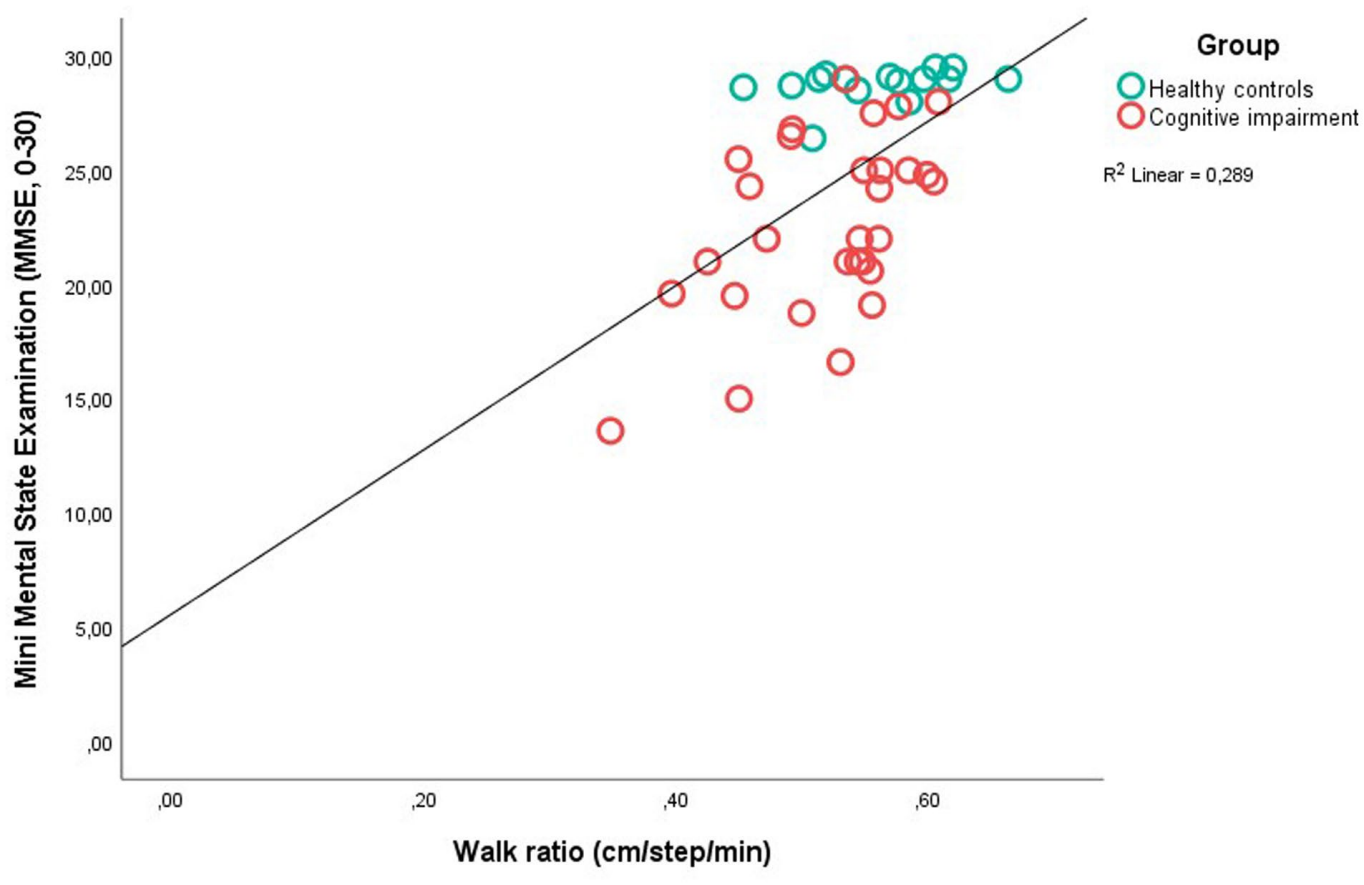
Scatter plot between Mini Mental State Examination and Walk ratio in patients with Cognitive Impairment and Healthy Controls.

**Figure 3:**
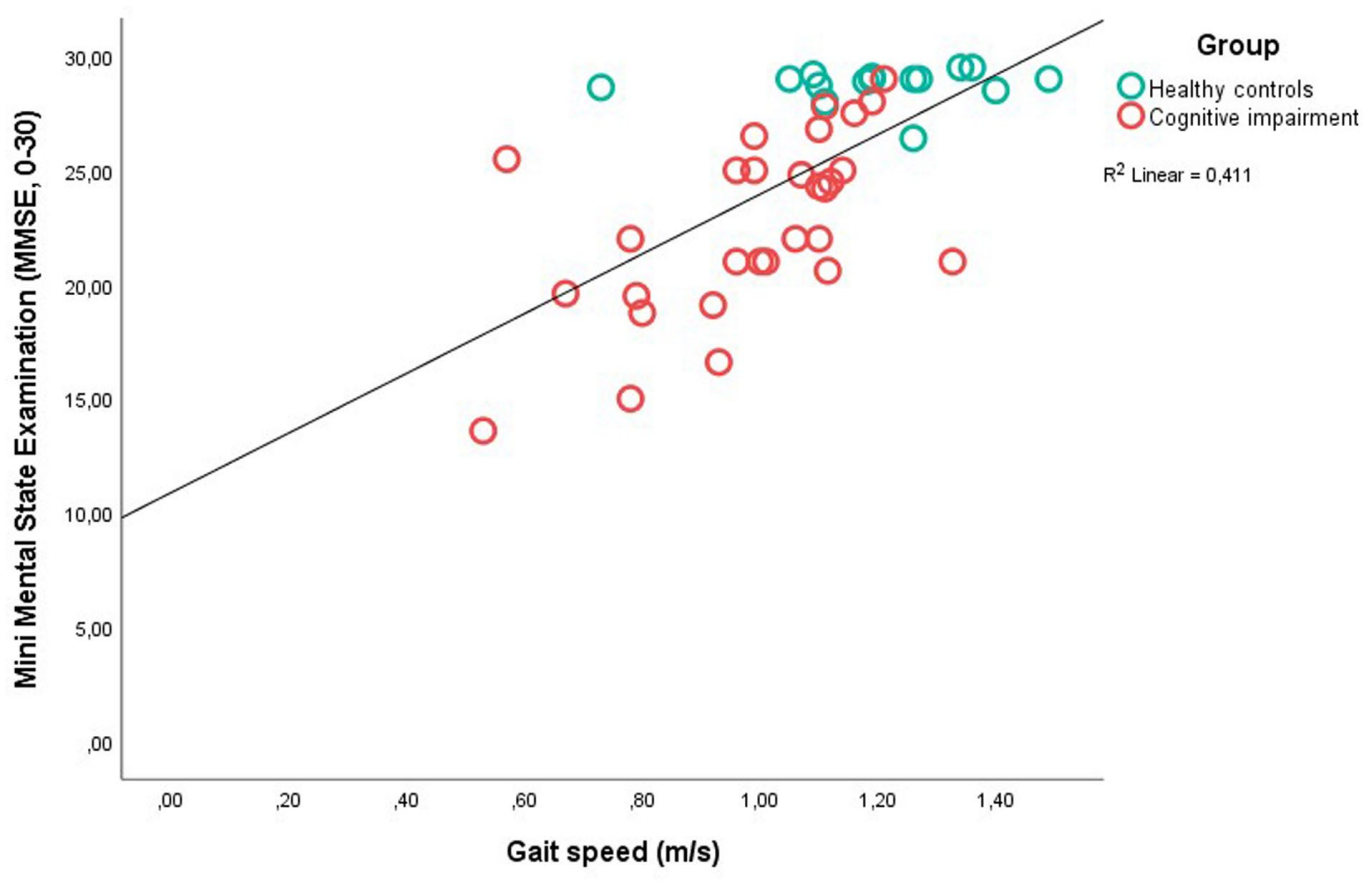
Scatter plot between Mini Mental State Examination and Gait speed in patients with Cognitive Impairment and Healthy Controls.

There was a bivariate correlation (Pearson’s *r*) between the Walk ratio and gait speed of 0.69 (*p* ≤ 0.001) (not shown in any table). The univariate and multiple regressions are shown in Table 3. In the univariate analysis there was a significant association between MMSE, and Walk ratio (*β* = 0.54, *p* ≤ 0.001), gait speed (*β* = 0.64, *p* ≤ 0.001) and age (*β* = −0.50, *p* = 0.001). A scatterplot between MMSE and Walk ratio, and MMSE and gait speed is displayed in Figures 2, 3. The explained variance for the model with age, MMSE and Walk ratio was 0.39, and 0.51 for the model with age, MMSE and gait speed. In the multiple regression analysis with both gait speed, Walk ratio and age, only age (*β* = −0.32, *p* = 0.011) and gait speed (*β* = 0.48, *p* = 0.003) were significantly associated with MMSE (Table 3). In the final, multivariable model, VIF varied between 0.50 and 0.70 and tolerance between 1.43 and 2.00, suggesting little collinearity.

**Table 3:** Linear regression with Mini Mental State Examination as dependent variable.

Independent variable	Beta	Coef	95% CI	Value of *p*	*R* ^2^
Unadjusted model					
WR	0.54	36.02	18.41 to 53.63	≤0.001	0.289
Gait speed	0.64	13.07	8.20 to 17.95	≤0.001	0.411
Age	−0.50	−0.517	−0.80 to −0.24	0.001	0.250
Adjusted for age model					
WR	0.41	27.32	9.57 to 45.07	0.003	0.393
Gait speed	0.54	10.96	6.23 to 15.69	≤0.001	0.511
Full model					
WR	0.09	5.69	−15.54 to 26.90	0.592	
Gait speed	0.48	9.86	3.56 to 16.16	0.003	
Age	−0.32	−0.33	−0.58 to −0.08	0.011	0.514

WR, Walk ratio; Beta, standardized beta coefficient; Coef, beta coefficient; CI, confidence interval of the beta coefficient.

## Discussion

In this literature review, we found that there was a stronger relationship between preferred gait speed and cognitive function (as measured with the MMSE), than between the Walk ratio and cognitive function. This suggests that preferred gait speed is a better indicator of cognitive functioning than the Walk ratio.

The Walk ratio was significantly lower in older adults with cognitive impairment than the healthy controls (Table 3), and lower than what is considered normal for healthy individuals (approximately 0.6) (Sekiya et al., 1996; Zijlstra et al., 2008; Bogen et al., 2018). In addition, there was an association between MMSE-scores and Walk ratios (*β* = 0.54, *p* = ≤0.001, *R*^2^ 0.289) (Table 3). The relationship between step length and step frequency is assumed to be invariant across different speeds and walking conditions, as this is the most efficient and least attention-demanding way of moving. Therefore, deviations from this relationship (lower or higher than approximately 0.6 cm/step/min) could suggest central nervous pathology (Egerton et al., 2011; Kalron et al., 2019), or that the context of the task requires attention, such as dual task-walking (Bogen et al., 2018). Lower Walk ratios, such as those found in the studies of people with dementia in this paper would typically involve shorter steps and higher step frequency, which can be interpreted as a cautious strategy, with shorter single support and swing phases (Zijlstra et al., 2008). It could be speculated that such a strategy is used when the automaticity of gait is compromised, for example in the presence of central nervous pathology (Clark, 2015). Early detection of cognitive impairment is important, and gait parameters can potentially be important in the diagnostic process (Montero-Odasso, 2016; Buckley et al., 2019). The Walk ratio is an interesting option, as it can be informative about gait parameters without need for digital devices, such as electronic walkways or body-worn sensors. However, in this study, the Walk ratio was less closely associated with MMSE than gait speed. In a multivariable regression model with both gait speed, Walk ratio and age, the Walk ratio was no longer significantly associated with MMSE (*β* = 0.09, *p* = 0.592), while gait speed (*β* = 0.48, *p* = 0.003) and age (*β* = −0.32, *p* = 0.011) were (Table 3). Gait speed relies on the functioning of most if not all bodily systems; joints must have appropriate range of motion, muscles must be strong enough and shut on and off at right times, senses must work for orientation and the metabolic systems must supply energy. Accordingly, if one or several systems are not working optimally, it tends to become apparent in how fast an older person walks (Studenski, 2009). In earlier studies, gait speed has been shown to predict dementia (Beauchet et al., 2016), to differ between dementia subtypes (Sverdrup et al., 2021) and is recommended in early detection of dementia (Montero-Odasso et al., 2020). In line with these findings, subtle alterations in the gait rhythm, such as those detected with the Walk ratio, appear to not be superior as an indicator of cognitive impairment.

The Walk ratio has been suggested as being invariant of all but very slow gait speeds (Sekiya et al., 1996; Murakami and Otaka, 2017), meaning that the relationship between step length and step frequency is the same whether a person walks at 0.9 meters/s or 2.0 meters/s. This makes the Walk ratio interesting, because many other gait parameters, such as between-step/stride variability or trunk accelerations vary according to gait speed, making adjustments for speed necessary (Moe-Nilssen, 1998). However, in our study, there was a correlation between gait speeds and Walk ratios, which counters this point (*r* = 0.69, *p* ≤ 001). This could suggest that the Walk ratio tends to be more speed-dependent than previously found. Alternatively, several of the included studies had average gait speeds that were quite low [for example, 0.57 m/s in Cosentino et al., 2020 and 0.67 m/s in Ijmker and Lamoth, 2012], which is near where the Walk ratio constancy started to break in Murakami and Otaka (2017) study. As such, earlier studies showing invariance of the Walk ratio (Zijlstra et al., 2008; Bogen et al., 2018).

In the included studies, only gait at preferred speed were used for analysis. There is ample evidence that older adults with cognitive impairment find complex walking more challenging than healthy older adults (De Cock et al., 2017). Making gait more complex can be achieved by adding for example cognitive tasks, such as backwards counting and memory tasks (Bridenbaugh and Kressig, 2011). In healthy older women, Zijlstra and co-authors found that the Walk ratio decreased while walking when carrying a tray and when carrying a tray and performing a Stroop task (Zijlstra et al., 2008), and in an earlier study, we found that the Walk ratio increased while counting backwards with intervals of three, but not during fast walking or uneven surface walking (Bogen et al., 2018). These findings imply that divided attention may have an impact on the Walk ratio, possible due to changes towards less automaticity and more reliance on compensatory executive control. It is therefore possible that other walking conditions would affect the results.

Further, in this study, cognitive functioning was operationalized as scores on the MMSE. The MMSE is well-established as a test for global cognition but is criticized among other things for its lack of sensitivity to cognitive impairment (Mitchell, 2009; Carnero-Pardo, 2014). Other measurement instruments that are directed at different cognitive domains, such as executive functioning, memory or verbal fluency would possibly give a better reflection of cognitive impairments in older adults.

## Limitations

The method that we used to estimate the Walk ratio is novel and not without limitations. The data were taken from group means, and not individuals, leading to uncertain values since the mean of the ratios of two variables is not equal to the ratio of their means. This also means that all studies have equal weight in the analyses, regardless of sample size. Further, we are comparing samples from different study designs. While this may not have had a great impact on the gait measurements, it can be assumed that the recruitment basis was different for the different studies. As such, there may be substantial heterogeneity in the included populations that is not brought forward, and the findings should be interpreted in this light. Finally, to reach firm conclusions about how people with cognitive impairment walk, more information about the participants than what we extracted from the included studies is necessary. For further studies, for example an embodied cognition perspective about the dynamic interplay between the person with cognitive impairment and the immediate surroundings could be explored. This is emphasized by previous research showing that people with cognitive impairment can have reduced spatial navigation abilities (Tangen et al., 2015; Lobo et al., 2018).

## Conclusion

In this literature review, we calculated the Walk ratio (cm/step/min) by dividing averages of step lengths by averages of step frequencies in older adults, with and without cognitive impairment. Both the Walk ratio and gait speed were significantly associated with MMSE on their own, but in multivariate regression with both Walk ratio and gait speed, only gait speed was significantly associated with MMSE. This suggests that gait speed should be the preferred gait parameter in the assessment of older adults with cognitive impairment.

## Author contributions

MK and IN had the idea for the study and carried out the initial analyses. BB contributed to further analyses. All authors participated in writing of the manuscript.

## Conflict of interest

The authors declare that the research was conducted in the absence of any commercial or financial relationships that could be construed as a potential conflict of interest.

## Publisher’s note

All claims expressed in this article are solely those of the authors and do not necessarily represent those of their affiliated organizations, or those of the publisher, the editors and the reviewers. Any product that may be evaluated in this article, or claim that may be made by its manufacturer, is not guaranteed or endorsed by the publisher.
