# Supplementary material for: The association between cognitive impairment, gait speed, and Walk ratio
Source: Front Aging Neurosci. 2023 May 18;15:1092990. doi: 10.3389/fnagi.2023.1092990 (PMC10233044; doi:10.3389/fnagi.2023.1092990)
Supplement: Supplementary file 1 [file Data_Sheet_1.docx]

Oppdatert litteratursøk

Gjennomført 17. oktober 2022

Av Gøril T. Jorem, HVL Biblioteket

Database: Ovid MEDLINE(R) and Epub Ahead of Print, In-Process, In-Data-Review & Other Non-Indexed Citations and Daily <1946 to October 14, 2022>

Search Strategy:

--------------------------------------------------------------------------------

1 (((step length or stride length) and (cadence or step frequency)) or ((gait or walk) adj1 (speed or velocity)) or walk ratio).tw. (9760)

2 exp Dementia/ (196040)

3 Cognitive Dysfunction/ (31952)

4 (dement* or alzheimer* or mild cognitive impairment).tw. (262395)

5 2 or 3 or 4 (317713)

6 1 and 5 (634)

7 limit 6 to yr="2021 -Current" (181)

***************************

Database: Embase <1974 to 2022 Week 41>

Search Strategy:

--------------------------------------------------------------------------------

1 (((step length or stride length) and (cadence or step frequency)) or ((gait or walk) adj1 (speed or velocity)) or walk ratio).tw. (14289)

2 exp dementia/ (415426)

3 mild cognitive impairment/ (33623)

4 (dement* or alzheimer* or mild cognitive impairment).tw. (361159)

5 2 or 3 or 4 (489068)

6 1 and 5 (928)

7 limit 6 to yr="2021 -Current" (171)

***************************

Database: AMED (Allied and Complementary Medicine) <1985 to September 2022>

Search Strategy:

--------------------------------------------------------------------------------

1 (((step length or stride length) and (cadence or step frequency)) or ((gait or walk) adj1 (speed or velocity)) or walk ratio).tw. (1392)

2 exp Dementia/ (3223)

3 Mild Cognitive Impairment/ (43)

4 (dement* or alzheimer* or mild cognitive impairment).tw. (4278)

5 2 or 3 or 4 (4278)

6 1 and 5 (20)

7 limit 6 to yr="2021 -Current" (2)

***************************

Database: APA PsycInfo <1987 to October Week 2 2022>

Search Strategy:

--------------------------------------------------------------------------------

1 (((step length or stride length) and (cadence or step frequency)) or ((gait or walk) adj1 (speed or velocity)) or walk ratio).tw. (1624)

2 exp Dementia/ (86141)

3 (dement* or alzheimer* or mild cognitive impairment).tw. (114266)

4 2 or 3 (115617)

5 1 and 4 (218)

6 limit 5 to yr="2021 -Current" (43)

***************************

Cochrane library


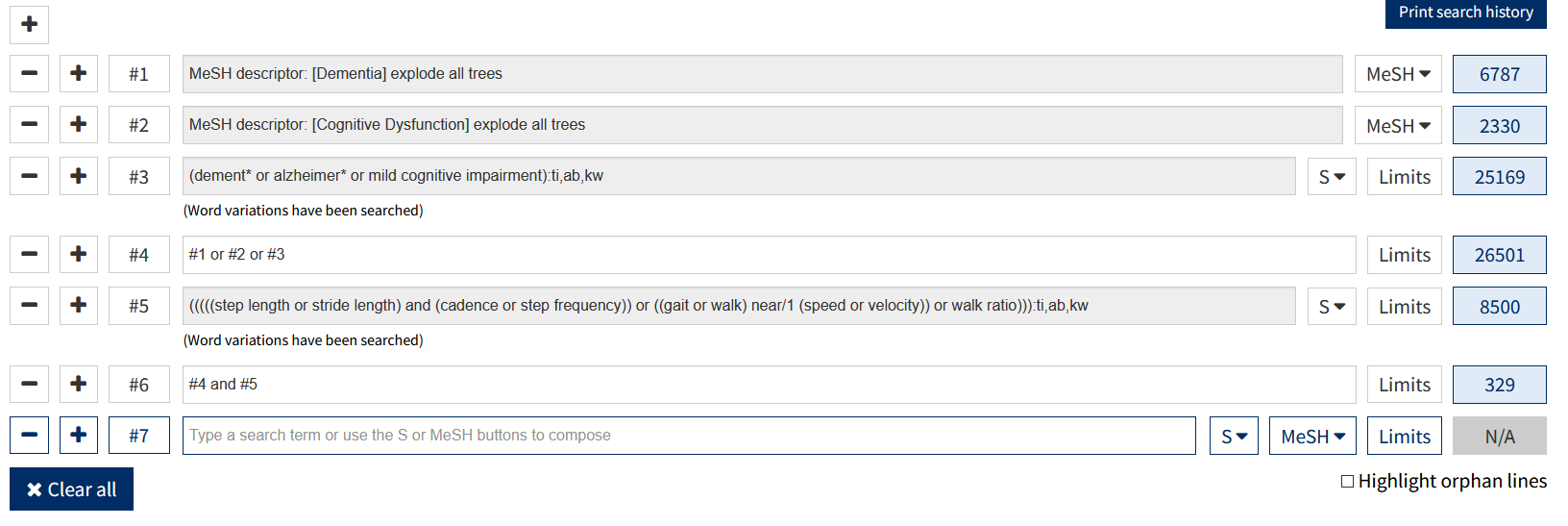


Avgrenset til utgivelser fra 1. januar 2021 og frem til i dag

PEDro

Kombinasjoner:

"step length" cadence dement*

"step length" cadence alzheimer*

"step length" cadence "mild cognitive impairment"

stride length AND cadence dement

stride length AND cadence alzheimer*

stride length AND cadence "mild cognitive impairment

step length AND step frequency dement*

step length AND step frequency alzheimer*

step length AND step frequency "mild cognitive impairment"

stride length AND step frequency dement*

stride length AND step frequency alzheimer*

stride length AND step frequency "mild cognitive impairment"

gait speed dement* (6)

gait speed alzheimer* (2)

gait speed "mild cognitive impairment" (3)

gait velocity dement*(1)

gait velocity alzheimer*

gait velocity "mild cognitive impairment"

walk speed dement*

walk speed alzheimer*

walk speed "mild cognitive impairment"

walk velocity dement*

walk velocity alzheimer*

walk velocity "mild cognitive impairment"

walk ratio dement*

walk ratio alzheimer*

walk ratio "mild cognitive impairment"

Total 12
